# Role of *Nannochloropsis Oculata* supplement in improving performance, antioxidant status, blood metabolites, and egg quality of laying hens under hot environmental conditions

**DOI:** 10.1038/s41598-024-66595-9

**Published:** 2024-07-23

**Authors:** K. R. S. Emam, Safaa A. M. Ali, A. S. Morsy, Wafaa A. Fouda, Ahmed M. Elbaz

**Affiliations:** 1 Animal and Poultry Production Department, Faculty of Agriculture, Beni-Suef University, Beni Suef, Egypt; 2 Animal and Poultry Physiology Department, Desert Research Center, Cairo, Egypt; 3 Animal and Poultry Nutrition Department, Desert Research Center, Cairo, Egypt

**Keywords:** Laying hens, Microalgae, Performance, Serum metabolites, Hot environmental conditions, Animal behaviour, Animal physiology

## Abstract

The increase in environmental temperature led to economic losses in the poultry industry, urging the use of feed supplements to mitigate the negative effects on chick's welfare and performance. Therefore, this study aimed to examine the effects of marine microalgae (*Nannochloropsis Oculata, N. Oculata*) additive on commercial Brown Lohmann laying hen's performance, blood metabolites, and antioxidant status under hot environmental conditions. One hundred and eighty birds (34 weeks old with an initial body weight of 1885 ± 23.5 g) were used till 47 weeks. The birds were divided into three equal groups (birds in each group were distributed into four equal replicates 15 hens/ replicate). The 1st group was the control (CON) and was fed the basal diet, while the 2nd (TR1) and 3rd (TR2) groups were fed the basal diet supplemented with 0.5 and 1.0% of *N. Oculata*, respectively. The results showed that total protein and globulin concentrations increased (P < 0.05) in treated groups compared with the control group, whereas, albumin concentration increased (P < 0.05) in TR2 compared to the control group. The concentration of ALT and AST decreased in hens fed *N. Oculata*. Supplementing with *N. Oculata* reduced serum cholesterol and creatinine concentrations, while glucose concentration increased (P < 0.05) in the treated groups compared to the control group. Serum calcium, total antioxidant capacity (TAC), Triiodothyronine (T3), and progesterone increased (P < 0.05) in hens fed *N. Oculata*. *N. Oculata* supplement improved production performance through a positive effect on egg number, egg weight, egg mass, feed conversion ratio, and mortality rate. In addition, the overall mean of shell thickness increased (P < 0.05) in hens fed *N. Oculata*. It can be concluded that the supplementation of 1.0% *N. Oculata* to the laying hens' diet enhanced productive performance, serum constituents, and antioxidant status under hot environmental conditions.

## Introduction

Heat stress is a major concern in the poultry industry, heat stress causes negative effects on feed intake, body weight gain, mortality, and other important traits governing the economic success of the poultry industry^1^. The high environmental temperature during the summer months is a major challenge for the breeder and layer industry in Egypt^2,3^. Environmental stressors, the most influential of which is heat stress, are particularly detrimental to the poultry industry^4,5^. The issue of environmental stress has become a great point of the world, especially in rearing animals. Heat stress results from a negative balance between the amount of heat energy produced by the bird and the net amount of energy flowing from the bird’s body to its surrounding environment. Therefore, stress is a biological response of the birds to the stimulus that negatively impacts their normal physiological equilibrium and welfare. Many studies have confirmed that heat stress negatively affects the performance of laying hens through the decrease in body weight, egg production, and egg weight, in addition to the deterioration of eggshell quality via stimulating a sequence of drastic changes that leads to an increase in reactive oxygen species ROS which leads to the deterioration of production performance^6,7^. Feed additives, including microalgae, play an important role in decreasing the negative effects of heat stress^8,9^, via their characteristics, including anti-inflammatory, anti-cancer, antimicrobial, and antioxidant properties, that will be revealed in this study.

*Nanoclolopsis Oculata* (*N. Oculata*) is a unicellular microalgae that can be grown in fresh and marine aquatic systems and is a known good source of proteins, minerals, phytopigments, and vitamins^10,11^. The high contents of various nutrients in microalgae made it a promising feed additive in poultry diets. Furthermore, microalgae contain numerous bioactive components in high amounts, such as flavonoids, β-carotene, phenolic acids, phycocyanins, steroids, saponin, chlorophyll, and triterpenoid^12^, which play an important role as an antioxidant, antiviral, anti-inflammatory, hepatoprotective, immune-stimulator, and antimicrobial^9,13,14^. Antioxidants may contribute to maintaining poultry health, preventing certain diseases, and alleviating the effects of heat stress. In addition, microalgae have many potential health-promoting effects, such as decreasing blood pressure and cholesterol, immune-stimulating, and promoting the growth of beneficial microorganisms in the intestine^15^, resulting in enhancing productive performance. A large and growing body of studies has revealed the immune-stimulatory, anti-inflammatory, antimicrobial, antiviral, and ant-oxidative activities of microalgae via enhancing disease resistance, stimulating antibodies and cytokines production, effectively scavenging free radicals, and inhibiting lipid peroxidation, thereby improving poultry production^14,16^. Additionally, the possibility of using algae as an alternative source of traditional protein (soybean meal) in poultry diets^9^. As a result of the many advantages confirmed by many previous reports, it was assumed that the addition of microalgae may reduce the harmful effects of heat stress on laying hens. Therefore, the current study aimed to investigate the effects of marine microalgae (*N. Oculata*) additive on commercial Brown Lohmann laying hen's performance and blood metabolites under hot climatic conditions.

## Results

### Thermo-respiratory responses

**Figure 1:**
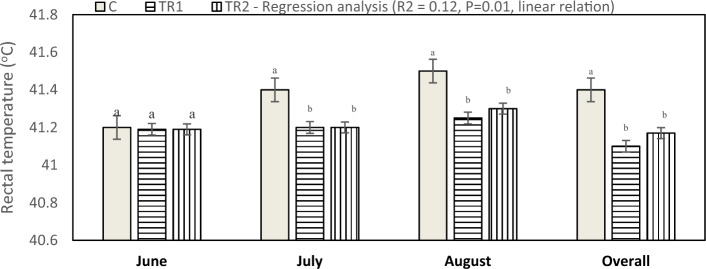
Effect of dietary supplementation with *N. Oculata* on body temperature (P = 0.001) of laying hens under hot environmental conditions. C, control group, birds fed a corn-soybean basal diet without, *N. oculata*; TR1, birds fed the basal diet supplemented with 0.5% of *N. oculata*; TR2, birds fed the basal diet supplemented with 1.0% of *N. oculata*. ^a–c^Mean value above each bar with no common superscript differs significantly (p < 0.05). Error bars represent SM.

**Figure 2:**
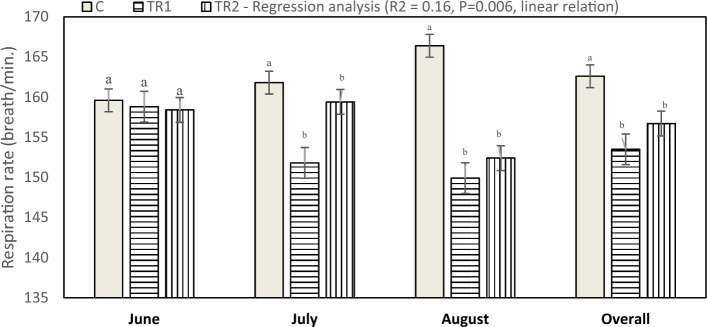
Effect of dietary supplementation with *N. Oculata* on respiration rate (P = 0.0031) of laying hens under hot environmental conditions. C, control group, birds fed a corn-soybean basal diet without, *N. oculata*; TR1, birds fed the basal diet supplemented with 0.5% of *N. oculata*; TR2, birds fed the basal diet supplemented with 1.0% of *N. oculata*. ^a–c^Mean value above each bar with no common superscript differs significantly (p < 0.05). Error bars represent SM.

In the current study, the average indoor ambient temperature (AT, ºC), relative humidity (RH, %), and temperature humidity index (THI) throughout the experimental period are presented in Table 1. The lowest mean AT (28.05 °C) was recorded in June, while the highest (31.35 °C) was observed in August. The overall means of THI refer to absent heat stress during the first month of the experiment, while, the experiment was exposed to moderate heat stress during July and August. Figures 1 and 2, showed that, during the first month of the experiment, there were insignificant differences observed between the treated groups and the control group in body temperature (BT) and respiration rate (RR), while, during the second (July) and third months (August) of the experiment, BT and RR significantly decreased in TR1 and TR2 groups compared with the control group.

**Table 1:** Average monthly indoor of ambient temperature (AT), relative humidity (RH) and temperature-humidity index (THI) during experimental period.

Month	AT (ºC)	RH (%)	THI
June	28.05	49.61	27.55
July	30.28	46.90	27.85
August	31.35	54.17	28.85
Overall mean	29.89	50.23	28.09

### Serum proteins

The effect of supplementing with *N. Oculata* on total proteins (TP), albumin, globulin concentrations, and albumin/ globulin (A/G) ratio of laying hens under hot climatic conditions is shown in Table 2. Results indicated that TP and globulin concentrations increased significantly (P < 0.05) in laying hens fed *N. Oculata* compared with the control group, while, albumin concentration increased significantly (P < 0.05) in the TR2 compared to TR1 and control group. On the other hand, there are insignificant differences (P < 0.05) among experimental groups in the A/G ratio. In addition, there was a significant linear effect of increasing doses of *N. oculata* in the chicken diet (P < 0.05) on protein metabolism, which indicates that increasing the dose of microalgae supplements may increase total protein and albumin levels.

**Table 2:** Effect of dietary supplementation with *N. Oculata* on serum proteins of Commercial Browne Lohmann LSL laying hens under hot environmental conditions.

Items	Experimental groups			± SE	P-values	Reg. analysis		
	C	TR1	TR2			R^2^	P	Relation
Total protein (TP, g/dl)	5.48^b^	6.39^a^	6.09^a^	0.15	0.0003	0.15	0.007	linear
Albumin (A, g/dl)	3.53^b^	3.63^b^	3.95^a^	0.09	0.0058	0.08	0.053	linear
Globulin (G, g/dl)	1.95^c^	2.76^a^	2.14^b^	0.16	0.0014	0.05	0.126	–
A/G ratio	1.81	1.31	1.84	0.19	0.0900	–	–	–

*C* control group, birds fed a corn-soybean basal diet without *N. oculata*, *TR1* birds fed the basal diet supplemented with 0.5% of *N. oculata*, *TR2* birds fed the basal diet supplemented with 1.0% of *N. oculata.*

^a–c^Means followed by different letters in superscript in the same row differ significantly (P < 0.05). ± SM, standard error of means. Number of samples: 30 chickens (30n).

### Liver enzymes activity and lipid profile

The effect of dietary supplementation of *N. oculata* on serum alanine aminotransferase (ALT) and aspartate aminotransferase (AST) concentrations is shown in Table 3. Results indicated that ALT and AST enzymes decreased significantly (P < 0.05) in the TR2 and TR1 compared to the control group. Table 3 shows the effect of dietary supplementation with *N. Oculata* on cholesterol (CHO), creatinine (CRE), and glucose (GLU) concentrations of laying hens under hot climate conditions. It was observed that dietary supplementation of *N. Oculata* significantly reduced (P < 0.05) CHO and CRE concentrations, while serum GLU concentration increased significantly (P < 0.05) in laying hens compared to the control group. In addition, there was a significant linear effect of increasing doses *of N. oculata* in the hen's diet (P < 0.05), which indicates that increasing the dose of microalgae supplements may enhance liver health by decreasing secretion enzymes liver under heat stress, including ALT and AST.

**Table 3:** Effect of dietary supplementation with *N. Oculata* on some serum metabolites of Commercial Browne Lohmann LSL laying hens under hot environmental conditions.

Items	Experimental groups			± SE	P-values	Reg. analysis		
	C	TR1	TR2			R^2^	P	Relation
ALT (IU/L)	27.17^a^	25.70^b^	26.11^b^	0.31	0.0037	0.14	0.011	Linear
AST (IU/L)	42.36^a^	39.10^b^	35.72^c^	0.68	0.0001	0.40	0.001	Linear
CHO (mg/dl)	184.31^a^	158.01^c^	172.70^b^	2.74	0.0001	0.07	0.067	–
CRE (mg/dl)	1.35^a^	0.76^b^	0.93^b^	0.12	0.0002	0.06	0.083	–
GLU (mg/dl)	210.80^b^	233.73^a^	241.99^a^	7.14	0.0079	0.16	0.005	Linear

*C* control group, birds fed a corn-soybean basal diet without, *N. oculata*, *TR1* birds fed the basal diet supplemented with 0.5% of *N. oculata*, *TR2* birds fed the basal diet supplemented with 1.0% of *N. oculata*, *AST* aspartate aminotransferase, *ALT* alanine aminotransferase, *CHO* total cholesterol, *CRE* creatinine, *GLU* glucose.

^a–c^Means followed by different letters in superscript in the same row differ significantly (P < 0.05). ± SM, standard error of means. Number of samples: 30 chickens (30n).

### Serum calcium and phosphorus

**Figure 3:**
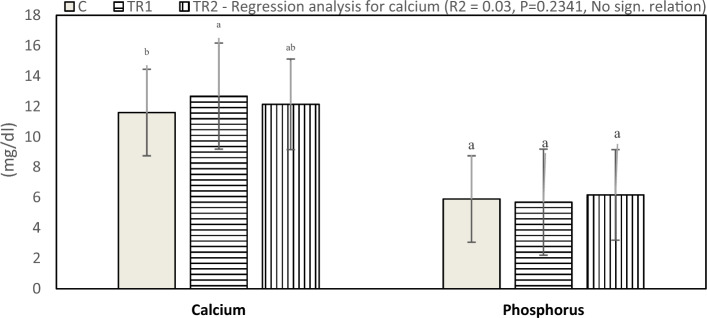
Effect of dietary supplementation with *N. Oculata* on calcium (P = 0.0024) and phosphorus levels (P = 0.3720) of laying hens under hot environmental conditions. C, control group, birds fed a corn-soybean basal diet without, *N. oculata*; TR1, birds fed the basal diet supplemented with 0.5% of *N. oculata*; TR2, birds fed the basal diet supplemented with 1.0% of *N. oculata*. ^a–c^Mean value above each bar with no common superscript differs significantly (p < 0.05). Error bars represent SM.

The effect of supplementation with *N. Oculata* on serum calcium (Ca) and phosphorus (P) concentrations is shown in Fig. 3. Results indicate that the Ca level increased significantly (P < 0.05) in the TR1 and TR2 groups compared to the control group. In contrast, the P level was not significantly affected (P < 0.05) between the experimental groups.

### Physiological stress indicators

**Figure 4:**
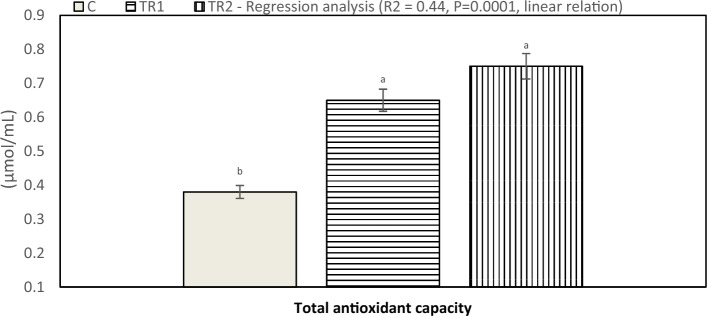
Effect of dietary supplementation with *N. Oculata* on total antioxidant capacity levels (P = 0.001) of laying hens under hot environmental conditions. C, control group, birds fed a corn-soybean basal diet without, *N. oculata*; TR1, birds fed the basal diet supplemented with 0.5% of *N. oculata*; TR2, birds fed the basal diet supplemented with 1.0% of *N. oculata*. ^a–c^Mean value above each bar with no common superscript differs significantly (p < 0.05). Error bars represent SM.

**Figure 5:**
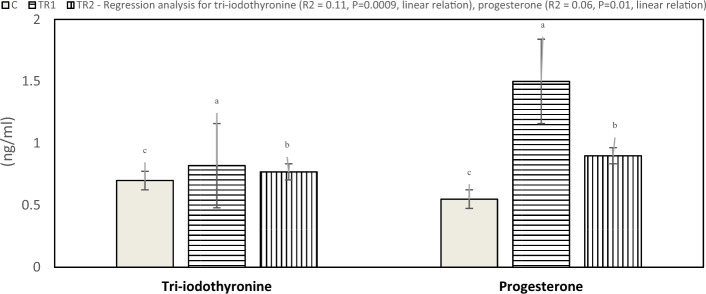
Effect of dietary supplementation with *N. Oculata* on tri-iodothyronine (P = 0.001) and progesterone levels (P = 0.001) of laying hens under hot environmental conditions. C, control group, birds fed a corn-soybean basal diet without, *N. oculata*; TR1, birds fed the basal diet supplemented with 0.5% of *N. oculata*; TR2, birds fed the basal diet supplemented with 1.0% of *N. oculata*. ^a–c^Mean value above each bar with no common superscript differs significantly (p < 0.05). Error bars represent SM.

**Figure 6:**
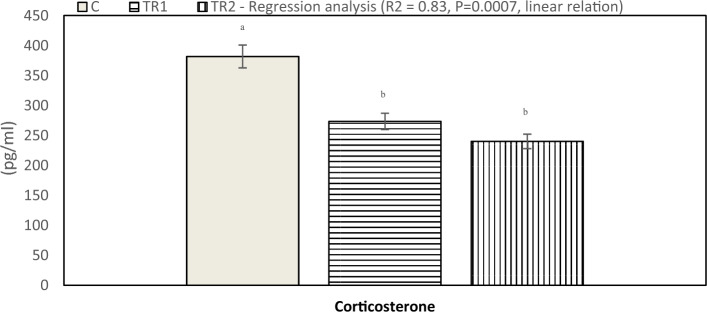
Effect of dietary supplementation with *N. Oculata* on corticosterone hormones levels (P = 0.008) of laying hens under hot environmental conditions. C, control group, birds fed a corn-soybean basal diet without, *N. oculata*; TR1, birds fed the basal diet supplemented with 0.5% of *N. oculata*; TR2, birds fed the basal diet supplemented with 1.0% of *N. oculata*. ^a–c^Mean value above each bar with no common superscript differs significantly (p < 0.05). Error bars represent SM.

Serum antioxidant response (TAC activity) affected by *N. Oculata* supplementation of laying hens under hot environmental conditions is presented in Fig. 4. Results indicated that hens fed a diet supplemented with 0.5 and 1.0% *N. Oculata* had significantly elevated TAC values (P < 0.05) compared to the control group. The effect of dietary supplementation of *N. Oculata* on serum tri-iodothyronine (T3) and progesterone (P4) concentration is presented in Fig. 5. The current study indicated that serum T3 concentration significantly increased (P < 0.05) in the TR1 and TR2 compared to the control group. Moreover, there was a significant increase (P < 0.05) in serum progesterone (P4) concentration for hens fed a diet supplemented with 0.5 and 1.0% *N. Oculata* compared to the control treatment. Furthermore, illustrated in Fig. 6, the laying hens in the TR1 and TR2 groups exhibited lower corticosterone levels compared with the control group during heat stress. The lowest corticosterone concentration was observed in the 1% *N. Oculata*-supplemented group under high ambient temperature.

### Productive performances

The effects of the supplementation of *N. Oculata* in the laying hen diets on productive performance indicators, including live body weight (LBW) and total weight gain (TWG), feed intake (FI), egg number (EN), egg weight (EW), egg mass (EM), feed conversion ratio (FCR) and mortality rate (MR), are presented in Table 4. It was observed that supplementing with *N. Oculata* had a positive effect (P < 0.05) on productive performances, except LBW and TWG, which were not affected by the experimental diets. Nevertheless, there was a significant improvement (P < 0.05) in EN, EM, EW, FCR, and mortality rate (MR), in laying hens fed N. oculata and those fed the control diet. Moreover, FI decreased significantly (P < 0.05) in laying hens fed a diet supplemented with *N. Oculata* compared to the control group. Interestingly, the best production performance was in hens that received 1.0% of *N. Oculata* (TR2) compared to the TR1 and control groups. A significant linear effect of increasing doses of *N. Oculata* was found (p < 0.05), which indicates that the higher the dose of microalgae supplementation, the improved the productive performances, including FI, EN, EW, EM, and FCR.

**Table 4:** Productive performances of Commercial Browne Lohmann LSL laying hens as affected by dietary additive with *N. Oculata* under hot environmental conditions.

Parameters	Experimental groups			± SE	P value	Reg. analysis		
	C	TR1	TR2			R^2^	P	Relation
Initial body weight (g)	1885	1886	1884	23.50	0.9982	–	–	–
Final body weight (g)	1897	1897	1896	15.23	0.2000	–	–	–
Daily feed intake/hen (g/day)	120.66^a^	105.01^b^	95.99^c^	1.67	0.0001	0.84	0.0001	linear
Total feed intake/hen (g)	12066^a^	10501^b^	9599^c^	167.25	0.0001	0.84	0.0001	linear
Egg number/hen	79.25^c^	84.66^a^	82.25^b^	0.53	0.0001	0.39	0.0002	linear
Egg weight /hen (g)	65.30^b^	67.67^a^	68.14^a^	0.74	0.0267	0.20	0.0120	linear
Total egg mass/hen (g)	5191.35^b^	5728.94^a^	5604.51^a^	76.45	0.0001	0.40	0.0002	linear
Feed conversion ratio	2.32^a^	1.83^b^	1.71^c^	0.04	0.0001	0.80	0.0001	linear
Mortality rate (%)	4.0	1.0	0.0	–	–	–	–	–

*C* control group, birds fed a corn-soybean basal diet without, *N. oculata*, *TR1* birds fed the basal diet supplemented with 0.5% of *N. oculata*, *TR2* birds fed the basal diet supplemented with 1.0% of *N. oculata.*

^a–c^Means followed by different letters in superscript in the same row differ significantly (P < 0.05). ± SM, standard error of means. Number of samples: 30 chickens (30n).

### Egg quality

Effect of dietary supplementation of *N. Oculata* on egg quality characteristics, including shell weight (ShW), shell percentage (ShP), shell thickness (ShTh), albumen weight (AW), yolk weight (YW), and yolk percentages (YP), are presented in Table 5. The obtained results indicated that dietary supplementation with 0.5 or 1.0% of *N. Oculata* did not affect (P < 0.05) ShW, while ShP. ShTh, AW, and YP significantly increased (P < 0.05) in the treated groups (0.5 and 1.0% N. oculata) compared to the control group. Regarding yolk weight (YW), the results indicated that hens fed 0.5% *N. Oculata* was not affected compared to the control group, while it significantly decreased (P < 0.05) in hens fed 1.0% *N. Oculata*. In addition, there was a significant linear effect of increasing doses of *N. oculata* in the hen's diet (P < 0.05), which indicates that increasing the dose of microalgae supplements may enhance egg quality, including EW, ShTh, AW, and YW.

**Table 5:** Egg quality traits of Commercial Browne Lohmann LSL laying hens as affected by dietary supplementation with *N. Oculata* under hot environmental conditions.

Parameters	Experimental groups			± SE	P value	Reg. analysis		
	C	TR1	TR2			R^2^	P	Relation
Egg weight (g)	61.80^b^	65.60^a^	67.03^a^	0.85	0.0001	0.17	0.0001	linear
Shell weight (g)	6.8	6.8	6.6	0.16	0.4600	–	–	–
Shell thickness (mm)	0.38^b^	0.43^a^	0.45^a^	0.02	0.0001	0.40	0.0001	linear
Shell (%)	11.00	10.40	9.85	2.11	0.0821	–	–	–
Albumen weight (g)	37.40^c^	40.83^b^	44.00^a^	0.83	0.0001	0.26	0.0001	linear
Albumen (%)	60.52	62.24	65.64	4.23	0.0932	–	–	–
Yolk weight (g)	17.57^a^	17.97^a^	16.50^b^	0.34	0.0068	0.05	0.0270	linear
Yolk (%)	28.43	27.40	24.61	3.25	0.1000	–	–	–

*C* control group, birds fed a corn-soybean basal diet without, *N. oculata*, *TR1* birds fed the basal diet supplemented with 0.5% of *N. oculata*, *TR2* birds fed the basal diet supplemented with 1.0% of *N. oculata.*

^a–c^Means followed by different letters in superscript in the same row differ significantly (P < 0.05). ± SM, standard error of means. Number of samples: 30 chickens (30n).

## Discussion

Heat stress is one of the biggest obstacles to the development of the poultry industry, which leads to deterioration in production performance and disruption of physiological processes through exposure to oxidative stress, poor feed utilization, and high mortality, which results in major economic losses. Nutritionists have used many feed additives in an attempt to mitigate the negative impact of heat stress on birds^9,17,18^. Therefore, the current study aimed to add marine microalgae (*N. Oculata*) to improve growth and physiological performance during heat stress. In our experiment, laying hens showed signs of heat stress, such as panting and wing straightening. The laying hens fed the *N. Oculata* also showed a lower body temperature (BT) and respiration rate (RR) than the birds fed the control diet. These results may be due to microalgae having many potential health-promoting effects, such as antioxidant activity, immune-stimulating properties, and promoting the growth of beneficial microorganisms in the intestine during heat stress^5,9,13^.

The health and performance of the bird are affected by physiological changes as a result of various environmental pressures, including the high environmental temperature. Our results showed a deterioration in production performance in birds fed a diet without additives (control group). Blood biochemical parameters are used as indications of the health status of the experimental birds. The present study focused on certain biochemical parameters related to liver and kidney functions, thyroid gland activity, antioxidant capacity status, and blood minerals such as calcium and phosphorus concentration.

The current results indicated that total proteins and globulin concentrations increased significantly in the groups fed *N. Oculata* compared with the control group, in addition, the increase in total proteins levels was accompanied by the increase in serum globulin concentration in hens fed *N. Oculata*, this may be related to the development of the immune system with the synthesis of immune globulin, thus, it's a good indicator of immunity response. These results are similar to Fathi et al.^19^ who reported that total proteins and globulin concentrations significantly increased in chickens fed supplemented with *Spirulina platensis* compared with the control group. Similarly, Abd El-Hamid et al.^11^ found that rabbits fed supplements of marine microalgae had elevated serum total proteins and globulin levels compared to the control group. Furthermore, Mariey, et al.^20^ found that adding *S. platensis* to the laying hen diets had a statistically positive effect on total proteins and globulin concentrations compared to the control group. On the contrary, Curabay et al.^21^ reported that there were insignificant effects when adding two levels of *S. platensis* (1 and 2%) to the laying hen rations on total proteins, albumin and globulin. Zeweil et al.^8^, and Elbaz et al.,^5^ found that *S. platensis* supplementation did not affect serum concentrations of total proteins, albumin, and globulin in broilers. These differences in the results of previous reports may be due to the concentration of microalgae and experimental conditions. This demonstrates the positive effect of microalgae supplements on protein metabolism in hen's hot environmental conditions.

Our results indicate that AST and ALT enzymes decreased significantly (P < 0.05) in groups fed *N. Oculata* compared to the control group during heat stress. Our results are in agreement with Abd El-Hamid et al.^11^ who studied the effect of supplementing the diet with *N. Oculata* on serum metabolites of Hi-Plus rabbits, which indicated that ALT and AST concentrations decreased in groups fed supplementation of *N. Oculata* compared with the control group. On the contrary, Abdel-Moneim et al.^9^ reported that serum concentrations of ALT and AST were not significantly affected by feeding on dietary supplements of *S. platensis* at levels of 5 and 10 g / kg compared to the control group in the broilers. The difference in the effect of algae on liver enzymes in previous studies may be due to the type of algae, the amount added, etc. In our study, however, the addition of *N. Oculata* algae may be considered to have an effective role in preserving the structural integrity of the hepatocellular membrane. When hepatocyte membranes are damaged, multiple enzymes normally located in the cytosol are released into the bloodstream^22^. Based on its antioxidant properties in algae, the therapeutic value may be gained by supplementing *N. Oculata* to protect the liver against oxidative injury in different stress conditions^23^.

Chicken are prone to lipid peroxidation due to physiological and dietary factors, resulting in increased peroxidative metabolites. Feeding lipid-rich diets to birds or exposure to heat stress may result in oxidative stress, which can damage DNA, bio-membrane lipids, proteins, and a range of tissue impairments^24^. Therefore, adding nutritional supplements such as algae enhances the antioxidant defense mechanisms within the liver and meat tissues through its antioxidant properties. In this study, It was observed that dietary supplementation of *N. Oculata* reduced significantly (P < 0.05) CHO, and CRE concentrations in groups that received *N. Oculata* compared to the control group. While chickens fed *N. Oculata* supplemented showed increased concentrations of serum GLU compared (P < 0.05) with control, in agreement with the present study^25^ when they found increased glucose (P ≤ 0.001) in chicken fed Spirulina-supplemented diets. Serum glucose is an important source of energy for poultry and can promote body tissue growth^24^. The results of the current study are consistent with Sarker et al.^26^ and Mariey et al.^20^ who reported that CHO levels significantly decreased in laying hens fed the *Spirulina*-containing diets. Similar results were obtained by Abouelezz^27^ who reported that the addition of Spirulina to the feed and/or to the drinking water of the Japanese quail had decreased significantly serum concentration CHO. The hypolipidemic effect of *Spirulina* has been reported to be due to the phycocyanin compound which inhibits pancreatic lipase activity and may be supplement dose-dependent^28^, in addition to bioactive compounds (phycocyanin and phycocyanobilin) that could alleviate the oxidative stress of kidneys by scavenging any free radicals and maintaining the normal values of creatinine and cholesterol as indicators to the healthy renal function. Moreover, some researchers attributed this decline in CHO to the content of polyunsaturated fatty acids in *Spirulina platensis* and the inclusion of newly formed CHO esters in HDL (high-density lipoprotein) by omega-3 fatty acids stimulating the lecithin cholesterol acyltransferase activity, this enzyme responsible for esterification of CHO^29^. Furthermore, Chen et al.^30^ reported that docosahexaenoic acid found in microalgae could inhibit the activity of 3-hydroxy3-methylglutaryl coenzyme A reductase by reducing CHO concentration. From these results, we conclude the effect of microalgae (*N. Oculata*) on enhancing the hen's health by improving lipid utilization in the blood, as evidenced by lower triglyceride and cholesterol levels.

Our results indicated that Ca levels increased significantly (P < 0.05) in groups that received *N. Oculata* compared to the control group, while serum P level was not affected (P < 0.05) among experimental groups. The results of the current study are similar to Abd El-Hamid et al.^11^ who found that Ca levels increased significantly in Hi-Plus rabbits fed *N. Oculata* compared to the control group. Many studies assert that microalgae have rich mineral content, which means that these plants can enhance the health of poultry and the product's quality. Additionally, the gut microbiota demonstrates a decrease in pathogenic microorganisms and an increase in beneficial microorganisms in chickens fed a diet containing algae supplements^31^, which enhance nutritional utilization and nutrient transportation.

Heat stress stimulates oxidative damage of tissues by increasing lipid peroxidation and corticosterone release in cell membranes, wherefore, serum corticosterone levels are commonly analyzed to screen for responses to stress stimuli in chickens^32^. Previous research has appreciated corticosterone levels as a stress index in chickens, which found that corticosterone increased in various tissues with stress^33^. In the current study, the serum corticosterone level decreased in groups fed *N. Oculata* during heat stress than the control group (P < 0.05), and changes in the corticosterone hormone levels may indicate the bird's exposure to stress. Many studies have confirmed the positive effect of feed additives to mitigate the harmful effects of heat stress, such as lowering corticosterone levels^34^. Similarly, supplementation of the diet with *Spirulina* resulted in lower secretion of corticosterone compared to the control group^35^. These results indicate that adding *N. Oculata* improves the bird's heat resistance may be due to the antioxidant properties of *N. Oculata*.

Oxidative stress is the considerable negative impact of heat stress, which impairs numerous metabolic dysfunctions and the performance of broilers^36^. Serum antioxidant capacity (TAC) enzyme activity is documented as an indicator of lipid peroxidation, and determining circulatory TAC is considered an indicator of total antioxidant capacity in maintaining the health of birds. Our results indicated that hens fed a diet supplemented with *N. Oculata* elevated TAC values more than the control group, which enhanced the oxidation status. Similar to our results, many previous studies have confirmed the enhancing role of feeding a diet containing microalgae on the oxidative stress status of birds^37,38^. Moreover, the role of dietary supplementation of *S. platensis* to enhance these antioxidative biomarkers of broilers under heat stress has been reported^9,37,39^. Several factors contribute to the anti-oxidative activities of microalgae as phycobiliproteins and biologically active compounds, including β-carotene, astaxanthin, lutein, bioactive peptides, phenolic compounds, phycocyanin and sulfated polysaccharides^37^, that serve as antioxidant agents that could neutralize the excessive free radicals and thus prevent oxidative damage^38^. Furthermore, Moradi et al.^40^ pointed out that supplementing with *Chlorella sp*. may increase selenium levels in the blood, which plays an important in improving the antioxidative status of chickens. Supplementing diets with algae upregulate antioxidant activities in tissues, contributing to boosted oxygen radical absorbance capacity and antioxidant enzyme activities^41^. Thus, supplementing with *N. Oculata* was able to restore redox equilibrium because of its bioactive antioxidant components.

Results of the current study showed an increase in the concentration of T3 and P4 in the serum of chickens fed diets containing *N. Oculata* compared to the control group. Consistent with our results, Abdel-Moneim et al.^9^ reported that supplementing with *Spirulina* at a level of 5 g/kg in the diet reduced levels of serum tri-iodothyronine hormone of heat-stressed chickens. Likewise, Fan et al.^42^ reported that T3 and thyroxine (T4) increased with the increase in the supplementation of brown seaweed (*Sargassum sp.)* meal in the diets of Leghorn layers. Our findings indicated an increase in egg laying from increased serum P4 levels, this indicated that dietary supplementation of *N. Oculata* led to increasing blood secretion of progesterone hormone and thus enhancing egg production (strong connection between laying eggs and serum P4 levels). In addition, this result reflects an improvement effect on egg production, egg weight, egg mass/hen, and feed conversion ratio and maintains the growth indices recorded for hens fed supplement with *N. Oculata* Consequently, dietary supplementation with *N. Oculata* actively plays a vital role in the functions and performance of the thyroid gland, as well as in keeping the metabolism and homeostasis stable and physiological performance normal.

The negative effect of heat stress on the birds is usually represented by many harmful physiological changes (the most important of which are oxidative stress and low blood acidity) and deterioration of intestinal integrity (imbalance in microbial composition and inflammation), which lead to a deterioration in productive performance. Therefore, we began our study by clarifying the effects of heat stress and experimental feed additives on the bird to explain their effect on the bird’s general performance. In the current study, live body weight and total weight gain were not affected by the experimental additives. However, it was noticed that the addition of *N. Oculata* had improved EN, EW, EM, FCR, and MR, and FI decreased significantly (p < 0.05) compared to the control group. Similar to our results, Curabay et al.^21^ found that *S. platensis* supplements did not affect the LBW of laying hens. Likewise, Sarker et al.^26^ studied the effects of the addition of *S. platensis* in laying hens diets and found no significant effect on TWG compared to the control hens. Moreover, Kharde et al.^43^ reported that the addition of 10% or above *S. platensis* to the diet could suppress the growth of poultry. Regarding the effect of algae on the productive performance of laying hens, our results agree with many studies that confirmed the positive role of adding algae in improving EN, EW, EM, and FCR^20,44^. Furthermore, Abu Hafsa et al.^45^ studied the effect of green and brown seaweed supplementation on laying performance and egg quality in Japanese quails and found that it had a positive effect on EN, EW, and EM, while feed intake was not significantly affected. In this study, a decrease in the mortality rate of birds fed microalgae reflects the improvement of the general health of birds. This result is supported by Cheong^46^ who noticed that supplementation of quails diet with 2% *Spirulina* resulted in a significant reduction in the mortality rate. Microalgae have high levels of micro and macro-elements and the ability to enhance the chicken's growth performance and feed efficiency^47^, owing to algae polysaccharides and protein properties that can increase the health and productivity of chickens. Furthermore, this improvement may have been caused by bioactive compounds and high amino acid digestibility in algae supplements^24^. We conclude from this the role of *N. Oculata* (microalgae) in supporting productive performance through enhancing the oxidation status, protein assimilation, and modifying blood lipids profile.

Regarding the effect of dietary supplementation of *N. Oculata* on egg quality characteristics, the obtained results indicated that dietary supplementation with *N. Oculata* enhanced shell percentage, shell thickness, albumen weight, and yolk percentages. This result is in agreement with Abbas et al.^48^ who found that shell thickness and shell percentage significantly increased in HY-Line W-36 commercial laying hens fed a diet supplemented with Spirulina platensis compared with the control group. Moreover, Fernandes et al.^49^ observed that YW significantly increased in Bankiva line laying hens fed supplemented with 0.50, 0.75, and 1% marine microalgae. The biological mechanism by which microalgae affected enhancing egg quality may be related to the abundant amino acids and minerals in the microalgae biomass. The improvement in performance characteristics may be related to the major role of microalgae *N. Oculata* in inducing a modulation in the gut microbial population that enhances the absorption of dietary vitamins and minerals and consequently increases feed utilization^20^, in addition to significantly increasing progesterone hormone (P4), which play an important role in reproductive functions in fowls^50,51^, including the development of reproductive organs, albumen synthesis, eggshell’s formation, and egg production^52^. Algae are characterized by their high content of essential vital nutrients such as protein, vitamins, minerals, essential fatty acids, and biologically active compounds making them beneficial for the overall health of the hens and a valuable addition to poultry feed, resulting in better egg production.

## Conclusion

The detrimental impacts of heat stress environmental on performance, antioxidant status, blood metabolites, and egg quality were significantly lower in laying hens fed *N. Oculata* supplements. This study demonstrated that supplementing the diet with 1.0% *N. Oculata* improved performance, enhanced the antioxidant status and thyroid activity of heat-stressed laying hens, and boosted egg quality. A linear impact of increasing doses of *N. Oculata* on the egg number, egg weight, egg mass, egg quality, protein metabolism, and feed conversion ratio was found, indicating that the higher the *N. Oculata* supplementation level, the better the productive performance under heat stress.

## Materials and methods

### Experimental design and bird management

The experiment was performed at a private poultry farm (Latitude 31° 29 N; Longitude 32° 34 E), North Sinai Governorate, in cooperation with the Animal and Poultry Production Division (Department of Animal and Poultry Physiology), Desert Research Center, Ministry of Agriculture and Reclamation, Cairo, Egypt, and Systel Telecom Company and Egyptian Center of Excellence for Bio-Saline Agriculture. A total number of one hundred and eighty healthy 34 weeks old of Commercial Browne Lohmann LSL laying hens at the peak of egg production stage with mean body weight 1885 ± 23.5 g were used till 47 weeks of age in the study. The birds were divided into three main equal experimental groups (birds in each group were distributed into four replicates with 15 hens replicate), The first experimental group served as the control group (C) which fed a corn-soybean basal diet without microalgae (*N. oculata*) while the second and third experimental groups were fed the basal diet supplemented with 0.5% (TR1) and 1.0% (TR2) of microalgae (*N. oculata*) respectively. Birds were housed in wire cages. Each cage was equipped with a linear feeder, and nipple drinkers, and all birds received feed and water ad libitum. All experimental diets were formulated based on NRC requirements^53^. The study lasted 13 weeks and was conducted during the summer season (From June to August months). The laying hens were fed a standard commercial diet (2690 kcal of ME/kg and 16.70% crude protein) and kept under a lighting regime of 16 h light (from 6:30 am to 10:30 pm) and 8 h dark. The chemical composition of microalgae (*N. Oculata*) is presented in Table 6. The experimental diet is described in Table 7. The chemical composition was determined according to the AOAC^54^ procedure. All animal care procedures were approved by the Ethics Commission on the research use of Farm Animals. Percentages of the cumulative mortality rate of laying hens of each experimental group were recorded throughout the experimental period (13 weeks).

**Table 6:** Chemical composition of *Nannochloropsis oculata* and quantitative constituents of minerals and amino acids profiles.

Chemical composition (g/100g)		Minerals profile (mg/100g)		Amino acids profile (mg/100g)			
Moisture	7.15	Fe	19.35	Methionine	69.52	Tyrosine	87.69
Crude protein	55.78	Zn	1.02	Cysteine	17.30	Threonine	39.21
Fat	6.61	Sodium	1862.70	Phenylalanine	16.24	Valine	50.36
Ash	12.29	Calcium	229	Lysine	15.20	Serine	11.64
Total carbohydrate	18.17	Potassium	798	Isoleucine	55.95	Glycine	9.98
		Magnesium	173	Leucine	65.11	Proline	31.52
				Aspartic acid	30.16	Alanine	20.24
				Glutamic acid	15.07	Arginine	8.56
				Histidine	13.22

**Table 7:** Composition and calculated analysis of the experimental layer diets.

Ingredients	Experimental groups		
	C	TR1	TR2
Crushed corn yellow	46.00	46.00	46.00
Soybean meal (48%)	21.00	20.50	20.00
Wheat	7.00	7.00	7.00
Barley	3.00	3.00	3.00
Wheat bran	8.75	8.75	8.75
Molasses	9.00	9.00	9.00
Limestone	0.40	0.40	0.40
*Nannochloropsis oculata*	0.00	0.5	1.0
Salt	2.00	2.00	2.00
Di-calcium phosphate	2.00	2.00	2.00
Vitamin-mineral premix*	0.40	0.40	0.40
Methionine	0.15	0.15	0.15
Lysine	0.15	0.15	0.15
Ethoxyquin	0.15	0.15	0.15
Analysis (g/kg dry matter basis)			
Dry matter, %	89.00	89.00	89.00
Crude protein, %	16.70	16.70	16.70
Crude fiber, %	3.60	3.60	3.60
Ether extracts, (%)	3.16	3.16	3.16
Ash, (%)	10.40	10.40	10.40
Ca (%)	2.65	2.65	2.65
P (%)	0.71	0.71	0.71
ME (Kcal/kg)	2690.00	2690.00	2690.00

*C* control group, birds fed a corn-soybean basal diet without Nannochloropsis oculata, *TR1* treatment 1, birds fed the basal diet supplemented with 0.5% of Nannochloropsis oculata, *TR2* treatment 2, birds fed the basal diet supplemented with 1.0% of Nannochloropsis oculata, *Ca* calcium, *P* phosphorus, *ME* metabolizable energy.

*Each kilogram contained: vitamin A 15,000IU; DL-α-tocopheryl acetate 30IU; cholecalciferol 15,000IU; menadione5.0 mg; thiamine 3.0mg; riboflavin 6.0 mg; niacin 20.0 mg; pyridoxine 5.0 mg; pantothenic acid 8.0 mg; folic acid 1.0 mg; vitamin b1 15 µg; M 80.0 mg; Z 60.0 mg; F 30.0 mg; Cu 5.0 mg and Se 0.15 mg.

#### Preparation of microalgae (*N. Oculata*)

The microalgae (*N. Oculata*) used in the current study were prepared and kindly provided by the Biotechnology Microalgae Culture Unit, National Research Center, Egypt. Microalgae were maintained in standard F/2 Guillards media^55^. The collected microalgae were stored in the refrigerator at 4 °C until the culture period was finished and then harvested by centrifugation. The different diet components were mixed with oil, including *N. Oculata*, extruded in the form of pellets, and stored in clean dried plastic bags at 4 °C until use.

#### Meteorological measurements

Ambient temperature (AT, °C) and relative humidity (RH, %) were recorded two times a week at 14.00 h (8 times monthly) using the digital hygro-thermometer instrument, while the temperature humidity index (THI < units) was calculated using the following equation according to Marai et al.,^56^. THI = db °C – [(0.31 – 0.31 × RH) × (db °C – 14.4)]. Where db °C = dry bulb temperature in centigrade, The THI values were classified as an absence of heat stress (< 27.8), moderate heat stress (27.8–28.8), severe heat stress (28.9–29.9), and very severe heat stress (> 30.0). The monthly averages of AT and RH were calculated and presented in Table 1.

### Biochemical and antioxidant capacity assays

At the end of the study, 5 ml of blood samples were collected from the wing vein (30 samples of each experimental group) and allowed to clot before being centrifuged at 3500 rpm for 20 min to separate clear serum samples, which were transferred into Eppendorf tubes (2.0 ml approximately) and stored at – 20 °C for determinations of total protein (TP, g/dl), albumin (Alb, g/dl), aspartate aminotransferase (AST, iu/l), alanine aminotransferase (ALT, iu/l), total cholesterol (CHO, mg/dl), glucose (GLU, mg/dl) and creatinine (CRE, mg/dl) and were determined using colorimetric techniques and the process outlined by Spinreact Co., Spain^57^. All of these assays were carried out spectrophotometrically on a Turner 690 Chemistry Analyzer. The serum globulin concentration was calculated by the difference between the concentrations of total protein and albumin. Serum inorganic phosphorus (SIP) concentration was determined according to Gomori^58^, while serum calcium concentration was carried out using the commercial kits in the auto analyzer device (DDS ® Spectrophotometric Kits, Diasis Diagnostic Systems Co., Istanbul, Turkey). The activity of total antioxidant capacity (TAC, µmol/mL) as a lipid peroxidation biomarker was assayed in serum samples using commercially available kits (Bio Diagnostic Research) according to Erel^59^.

### Hormone determination

Triiodothyronine (T_3_), and progesterone (P_4_), were analyzed using ELISA kits (Monobind, USA) according to Abraham^60^; and Wheeler et al.,^61^, respectively. The intra -and inter-assay CV's are 9.3 and 8.83, respectively. Serum corticosterone concentration was examined using a chicken corticosterone ELISA kit (Enzo Life Science, Farmingdale, New York, USA) according to the manufacturer's instructions.

### Thermo-respiratory responses

At 2.00 pm, fifteen hens per experimental group were used to measure both cloacal temperature (CT, °C) and respiratory rate (RR, breath/minute). Cloacal temperature as body temperature indicator was measured using a calibrated clinical thermometer (Model Incoterm, Porto Alegre /RS, Brazil with ± 0.1 °C accuracy), with a temperature range of 32–43.9 °C. To acquire each cloaca temperature Respiration rate (RR, breath/minute) was measured by counting the wave cycles of the breast up and down per 30 s, and the obtained value was multiplied by 2 to give the number of breaths per minute. Both CT and RR values were measured two times per week (8 times/ month) and the monthly averages of CT and RR were calculated in the three experimental groups.

### Productive performance

A total of 90 birds (30 of each experimental group) were weighed weekly in the early morning (08:00 h) on a digital scale at ± 0.1g precision to determine the changes in body weight throughout the experimental period (13 weeks). The experiment commenced when the hens were 22 weeks old and continued until 34 weeks. The initial, and final body weight and feed intake (g) were measured, and the total weight gain (TWG) and feed conversion ratio (FCR, g feed/g egg) were calculated. The hen's performance including hen-day egg production, feed intake, and egg weight (g/hen/day) was measured while egg mass was calculated by multiplying the egg numbers and egg weight (g) for different replicates within each group.

### Egg quality

A total of 90 eggs were randomly collected from the three experimental groups (30 eggs per group) to assess separately egg quality parameters. Before egg breaking, egg weight (EW, g) was measured using an electronic weighing scale. Using a plastic scraper, the albumen was separated from the yolk, and then albumen weight (AW, g) and yolk weight (YW, g) were individually weighed using an electronic weighing scale. The eggshells were carefully washed separately, air-dried, and weighed with a digital scale (ShW, g). Then, eggshell membranes were removed, and separately eggshell thickness (ShT, mm × 10^−2^) was measured at three regions (top, equator, and base) in a micrometer unit using a caliper according to Yoruk et al.^62^.

### Statistical analyses

The significance between the variables of groups was analyzed by a one-way analysis of variance (ANOVA) using General Linear Model Procedure^63^. Duncan’s New Multiple Range Test^64^ separated differences among treatment means. Given the increasing nature of microalgae doses, linear and quadratic polynomial contrasts were used to evaluate their effects on productive performances (Table 4). Differences were considered statistically significant when P < 0.05. All data were expressed as mean ± standard error of the mean (SEM). The mortality rate of hens was analyzed by Chi-square analysis.

### Ethics approval and consent to participate

This study was conducted in accordance with the Local Experimental Animals Care and Welfare Committee and approved by the Institutional Ethics Committee affiliated with the Desert Research Center. (Approval No. 2023-041). All protocols were carried out in accordance with the Universal Directive on the Protection of Animals Used for Scientific Purposes. All protocols follow the ARRIVE guidelines for reporting animal research (https://arriveguidelines.org).

## Data Availability

The datasets used and/or analyzed during the current study are available from the corresponding author upon reasonable request.

## Abbreviations

Alb: Albumin
AST: Aspartate aminotransferase
ALT: Alanine aminotransferase
CHO: Total cholesterol
CRE: Creatinine
C: Chicks fed a corn-soybean basal diet without *N. oculata*
EN: Egg number
EW: Egg weight
EM: Egg mass
FI: Feed intake
GLU: Glucose
LBW: Live body weight
TWG: Total weight gain
FCR: Feed conversion ratio
MR: Mortality rate
T3: Triiodothyronine
P4: Progesterone
TP: Total protein
THI: Temperature humidity index
TR1: Chicks fed the basal diet supplemented with 0.5% of *N. oculata*
TR2: Chicks fed the basal diet supplemented with 1.0% of *N. oculata*
